# Comprehensive Approach to Medical Nutrition Therapy in Patients with Type 2 Diabetes Mellitus: From Diet to Bioactive Compounds

**DOI:** 10.3390/antiox12040904

**Published:** 2023-04-10

**Authors:** Luigi Barrea, Claudia Vetrani, Ludovica Verde, Evelyn Frias-Toral, Florencia Ceriani, Simona Cernea, Annamaria Docimo, Chiara Graziadio, Devjit Tripathy, Silvia Savastano, Annamaria Colao, Giovanna Muscogiuri

**Affiliations:** 1 Dipartimento di Scienze Umanistiche, Università Telematica Pegaso, Via Porzio, Centro Isola F2, 80143 Napoli, Italy; 2 Centro Italiano per la Cura e il Benessere del Paziente con Obesità (C.I.B.O), Unità di Endocrinologia, Diabetologia e Andrologia, Dipartimento di Medicina Clinica e Chirurgia, Università degli Studi di Napoli Federico II, Via Sergio Pansini 5, 80131 Naples, Italy; 3 Department of Public Health, University of Naples Federico II, Via Sergio Pansini 5, 80131 Naples, Italy; 4 School of Medicine, Universidad Católica Santiago de Guayaquil, Av. Pdte. Carlos Julio Arosemena Tola, Guayaquil 090615, Ecuador; 5 Nutrition School, Universidad de la Republica (UdelaR), Montevideo 11100, Uruguay; 6 Department M3/Internal Medicine I, George Emil Palade University of Medicine, Pharmacy, Science, and Technology of Târgu Mures, 540146 Târgu Mureş, Romania; 7 Diabetes, Nutrition and Metabolic Diseases Outpatient Unit, Emergency County Clinical Hospital, 540146 Târgu Mureş, Romania; 8 Unità di Endocrinologia, Diabetologia e Andrologia, Dipartimento di Medicina Clinica e Chirurgia, Università degli Studi di Napoli Federico II, Via Sergio Pansini 5, 80131 Naples, Italy; 9 Division of Diabetes UT Health and ALM VA Hospital, San Antonio, TX 78229, USA; 10 Cattedra Unesco “Educazione Alla Salute e Allo Sviluppo Sostenibile”, Università degli Studi di Napoli Federico II, Via Sergio Pansini 5, 80131 Naples, Italy

**Keywords:** type 2 diabetes, diet, nutritionist, nutrients, nutraceuticals, functional foods

## Abstract

In the pathogenesis of type 2 diabetes mellitus (T2DM), diet plays a key role. Individualized medical nutritional therapy, as part of lifestyle optimization, is one of the cornerstones for the management of T2DM and has been shown to improve metabolic outcomes. This paper discusses major aspects of the nutritional intervention (including macro- and micronutrients, nutraceuticals, and supplements), with key practical advice. Various eating patterns, such as the Mediterranean-style, low-carbohydrate, vegetarian or plant-based diets, as well as healthy eating plans with caloric deficits have been proven to have beneficial effects for patients with T2DM. So far, the evidence does not support a specific macronutrient distribution and meal plans should be individualized. Reducing the overall carbohydrate intake and replacing high glycemic index (GI) foods with low GI foods have been shown as valid options for patients with T2DM to improve glycemic control. Additionally, evidence supports the current recommendation to reduce the intake of free sugars to less than 10% of total energy intake, since their excessive intake promotes weight gain. The quality of fats seems to be rather important and the substitution of saturated and trans fatty acids with foods rich in monounsaturated and polyunsaturated fats lowers cardiovascular risk and improves glucose metabolism. There is no benefit of supplementation with antioxidants, such as carotene, vitamins E and C, or other micronutrients, due to the lack of consistent evidence showing efficacy and long-term safety. Some studies suggest possible beneficial metabolic effects of nutraceuticals in patients with T2DM, but more evidence about their efficacy and safety is still needed.

## 1. Introduction

Type 2 diabetes mellitus (T2DM) is a multifactorial disease where different genetic and/or environmental (lifestyle) factors play a role in the pathogenesis [1,2].

As for genetic factors, more than 400 polymorphisms (i.e., the presence of two or more variant forms of a specific DNA sequence) have been associated with T2DM [3]. Indeed, genome-wide association studies (GWES) detected polymorphisms related to the response to dietary intake [4] and regulation of insulin secretion and sensitivity in target tissues [5,6], which might explain the 18% risk of T2DM [3].

The main lifestyle factors associated with T2DM are as follows:

- Diet. It has been long suggested that high-density energy/ultra-processed foods and Western-type diets are associated with an increased risk of T2DM, while healthy dietary patterns (such as the Mediterranean diet) are associated with a lower risk [7,8,9]. It is apparent that adherence to a diet rich in whole grains, nuts, legumes, and vegetables, as compared to one rich in refined carbohydrates (CHO), red and processed meat, and sugar-sweetened beverages, confers benefits in terms of reducing the risk of T2DM [8,10]. The quantity and quality of CHOs might be relevant for the risk of T2DM, as results from several cohort studies suggested that a higher CHO intake (>70% of total calories), higher dietary glycemic load and glycemic index, and a lower intake of dietary fiber are associated with an increased risk of diabetes [11,12,13]. Several recent meta-analyses indicated that a higher intake of vegetable fats/olive oil/linoleic acid could be beneficial for the prevention of T2DM, while long-chain omega-3 appears to have minimal or no effect on the likelihood of developing diabetes [14,15,16,17]. Regarding the micronutrients, data support the association between low serum levels of 25-hydroxyvitamin D and a higher risk of prediabetes and T2DM, as well as the role of vitamin D supplementation in reducing the risk of T2DM [18,19,20,21,22,23]. In addition, increased dietary magnesium intake seems to be associated with a reduced risk of diabetes [24,25]. Coffee and light-to-moderate alcohol consumption are inversely associated with the risk of T2DM, although the latter might increase the risk in individuals with non-alcoholic fatty liver disease (NAFLD) [26,27,28,29].
- Poor Physical Activity. A sedentary lifestyle and T2DM are very strongly correlated. A recent meta-analysis, incorporating data from 1,331,468 participants, has shown an increased risk for all-cause and cardiovascular diseases (CVD) mortality and incidence of T2DM with higher levels of total sitting as well as TV viewing time [30]. In contrast, cardiovascular health, body weight, glycemic control, and the development of diabetes-related long-term complications are positively influenced by physical activity [31,32]. The focus is mainly on aerobic exercise, consisting of continuous, rhythmic movement of large muscle groups, such as walking, jogging, and cycling. The most recent guidelines of the American Diabetes Association (ADA) recommend regular physical activity (200–300 min/week) [33]. Cardiac output is improved by moderate to vigorous (65–90% of maximum heart rate) aerobic exercise training, which is associated with significantly reduced cardiovascular and overall mortality risk in patients with T2DM [34]. Moreover, a meta-analysis (846 participants: 440 in the intervention group, and 406 in the control group) reported that regular exercise improved blood glucose control and enhanced insulin sensitivity in patients with T2DM compared with physically inactive patients [35].
- Smoking. There is a strong association between smoking and T2DM. Smoking leads to insulin resistance and/or inadequate compensatory insulin secretion through various underlying effects, including oxidative stress, inflammation, and endothelial dysfunction [36,37,38]. Nicotine in cigarettes may also exert a direct toxic effect on beta cell function [39]. In addition, although smoking tends to decrease weight, it leads to central adiposity, which is linked to inflammation and insulin resistance [40,41,42,43]. A meta-analysis comprising 88 prospective studies (with nearly 6 million participants and 295,446 incident T2DM cases) showed that various smoking behaviors (active and passive smoking and smoking cessation) are correlated with an increased risk of T2DM in a dose-dependent manner [44].

Other risk factors that may be influenced by diet have been shown to increase the risk of T2DM:

- Sleep quality. A U-shaped relationship between sleep duration and T2DM risk has been identified by 2 meta-analyses, with lowest risk at 7–8 h per day [45,46]. Stress, depression, lower socioeconomic status, environmental chemicals, and abnormal intrauterine milieu seem to be additional contributing factors that modulate the risk [47,48].
- Obesity. Weight gain, body fat distribution, and BMI are closely linked to T2DM. A primary risk factor in T2DM is the increase of adipose tissue, especially if this is accumulated in the central abdominal area. Adipocytes can secrete a variety of hormones and cytokines, namely tumor necrosis factor (TNF)-α, interleukin (IL)-6, and resistin, which are able to induce a chronic inflammatory state and insulin resistance, with consequences primarily on adipose, muscular, and hepatic tissues [49,50]. In this situation, insulin has markedly impaired anti-lipolytic effect. Consequently, there is an increased production and secretion of free fatty acids in the systemic circulation which is also responsible for insulin resistance. Furthermore, in patients with obesity and with metabolic syndrome, low adiponectin levels and leptin resistance are frequently observed [51,52,53]. Leptin, produced by adipocytes, is a hormone with an anorexigenic activity that aids in regulating energy balance by inhibiting hunger [54]. At the same time, adiponectin is a peptide synthesized by adipocytes that presents anti-inflammatory and anti-atherogenic effects, and it is also an insulin sensitizer [55]. Therefore, individuals with obesity may present a dysregulation of adipokine levels with an increased risk of overfeeding and cardiometabolic diseases.
- Microbiota Dysbiosis. Normally, the gut microbiota provides numerous beneficial functions for the human host. These include involvement in lipid and CHO metabolism, hormone modulation, protection against pathogens, epithelial cell proliferation, and synthesis of vitamins and amino acids [56,57,58,59]. Additionally, gut microbiota breakdown otherwise indigestible molecules, including plant polysaccharides and some human milk oligosaccharides [56,57,58,59,60]. Several studies identified that changes in the quantity and diversity of gut microbiota have a significant impact on the progression of many metabolic disorders [61,62]. These changes in the gut microbiota composition can reshape intestinal barrier functions and induce metabolic and signaling pathways related to insulin resistance [63].
- Polycystic Ovary Syndrome (PCOS). PCOS is a common hormonal disorder characterized by excessive androgen production by the ovaries, which causes irregular menstrual cycles, hirsutism, acne, and, frequently, obesity [64]. Many women with PCOS exhibit basal and glucose-stimulated hyperinsulinemia and are insulin resistant, independent of BMI [65,66]. Kakoly et al., in a recent meta-analysis including 40 studies, indicated that the incidence of impaired glucose tolerance and T2DM is higher in women with PCOS compared to women without PCOS [67].

Although all these lifestyle factors should be considered also for the management of T2DM to reduce disease progression and complications, medical nutritional therapy is the main cornerstone in the treatment of T2DM.

Therefore, the present review aimed to provide an updated and more practical evidence-based guideline for dietitians and nutritionists about the management of T2DM by medical nutritional therapy. In addition, the role of nutraceuticals/bioactive compounds was also discussed.

## 2. Medical Nutritional Therapy

Medical nutritional therapy plays a crucial role in the management of T2DM. The goals of nutritional therapy endorsed by the ADA 2022 [68] include the following:

- To promote healthy eating patterns and appropriate portion sizes, considering individual needs and personal and cultural preferences.
- To support the pleasure of eating but limiting food choices that have been associated with detrimental effects on health.
- To provide the patient (and caregivers) practical tools to achieve a healthier eating pattern and improve overall diet quality.
- To emphasize that the nutritional goals for patients with T2DM are similar to those recommended to the general population.

## 3. Eating Patterns

A variety of dietary patterns may be effective for managing T2DM [68,69]. The Mediterranean-style, low-CHO, vegetarian or plant-based, DASH (Dietary Approaches to Stop Hypertension), or macrobiotic diet are all favorable dietary patterns that have shown benefits for patients with T2DM [70,71,72,73,74,75,76,77]. For other dietary patterns (e.g., Paleo diet, very-low-fat diets, intermittent fasting, etc.) there is less consistent evidence, and data need to be substantiated. A recent meta-analysis of 7 studies (338 patients with T2DM) showed that intermittent fasting induced a greater weight loss, but no further reduction of HbA1c compared with a standard diet [78].

So far, no single eating pattern or meal composition has been identified as superior to the others. However, some common features have been proven to be beneficial (i.e., choosing non-starchy vegetables, reducing sugars and refined grains, and increasing whole foods consumption instead of highly processed foods) [79,80].

Modest persistent weight loss ameliorates glycemic control, blood pressure, and lipid profile and may minimize the need for medications to control these risk factors in patients with both T2DM and overweight or obesity [81,82]. Healthy eating plans that result in an energy deficit of 500–750 kcal/day, which in most cases means an intake of approximately 1200–1500 kcal/day for women and 1500–1800 kcal/day for men, can lead to beneficial effects in terms of glycemic control, lipid profile, and blood pressure [33]. The results of the Look AHEAD study support this approach [83]. The study included 5145 overweight/obese patients with recently diagnosed T2DM, randomized to an intensive lifestyle intervention (with the goal to obtain a weight loss of >7% by following a 1200–1800 kcal/day diet with reduced total and saturated fat and increased physical exercise) or the current standard of care for 4 years. It resulted in significantly greater weight loss (−6.15% vs. −0.88%, *p* < 0.0001), improved HbA1c (−0.36% vs. 0.09%, *p* < 0.0001) along with other cardio-metabolic parameters, and a greater proportion of patients with diabetes remission (7.3% vs. 2.0% at 4 years) in the intervention group vs. the control group [83].

Clinical benefits generally begin when achieving a ≥5% weight loss, and more intensive weight loss (i.e., 15%) may be appropriate to maximize benefits [33,84,85]. Furthermore, very-low-calorie diets, typically 800–1000 kcal/day, integrated with high-protein foods and meal replacement products, may increase the rate and/or amount of initial weight loss and glycemic improvements compared with standard behavioral interventions [81,82,83,84,85,86].

## 4. Macronutrients

Many studies have been carried out to define the proper macronutrient distribution. Evidence supports that, for all people with or at risk for T2DM, there is not an ideal percentage of calories from CHO, protein, and fat [33]. Therefore, macronutrient partition should be established based on the individualized assessment of current eating patterns, preferences (e.g., tradition, culture, religion, health beliefs and goals, economics), and metabolic goals to determine the best dietary plan [87,88].

### 4.1. Carbohydrates

CHOs are classified into three main groups by the degree of polymerization: sugars (monosaccharides: glucose, fructose, galactose; disaccharides: sucrose, lactose, maltose, trehalose; and polyols: sorbitol, mannitol, lactitol, xylitol, erythritol, isomalt, maltitol), oligosaccharides (short-chain CHOs comprise α-glucans: maltodextrins, and non-α-glucans: raffinose, stachyose, fructo- and galacto-oligosaccharides, polydextrose, inulin), and polysaccharides (long-chain carbohydrates divided into starch: amylose, amylopectin, modified starches; and non-starch polysaccharides: cellulose, hemicellulose, pectin, arabinoxylans, b-glucan, glucomannans, plant gums, mucilages, and hydrocolloids) [89]. After being digested and absorbed, CHOs are delivered to the circulation causing an elevation of the blood glucose, which stimulates insulin secretion by β-cells.

Consequently, as the primary goal for the management of T2DM is to achieve an adequate fasting and postprandial glycemic control, CHO intake needs to be primarily addressed. Most patients with T2DM report consuming a moderate amount of CHO (44–46% of total calories). However, lowering CHO intake through a low- (26–45% of total calories) or very-low-CHO diet (less than 26% of total calories) has been shown as a valid option for patients with T2DM to improve glycemia [88]. It should be noted though that very-low-CHO dietary patterns are not recommended for pregnant or lactating women, people with or at risk for eating disorders, or people who have renal disease, and they should be used with caution in patients taking sodium–glucose cotransporter (SGLT) 2 inhibitors due to the potential risk of ketoacidosis [90,91]. A recent meta-analysis of 23 studies (1357 participants, age range of 47 to 67 years) showed that patients with T2DM after 6 months of a low- and very-low-CHO diet have improved HbA1c < 6.5% and or fasting glucose levels [92]. In addition, patients had a more significant weight loss, a reduction in diabetes medication, and improved triglycerides levels and insulin resistance compared with control diets (which primarily were low-fat diets) [92].

However, efforts to modify usual dietary patterns are often unsuccessful in the long-term, as people generally tend go back to their usual macronutrient distribution. Thus, the recommended approach is to individualize dietary plans with a macronutrient distribution that is more consistent with personal preference and usual intake to increase the likelihood of long-term maintenance. Patients with T2DM are encouraged to reduce the consumption of refined CHOs and added sugars, and instead choose CHOs from vegetables, legumes, fruits, dairy (milk and yogurt), and whole grains [68].

#### 4.1.1. Glycemic Index and Glycemic Load

The utilization of the glycemic index (GI) was introduced by Jenkins D. et al. in 1981 as an instrument for patients with T2DM to select foods [93]. Foods containing CHOs have a different blood glucose-raising potential, defined as GI, resulting in a distinct glycemic response (GR). GR refers to the appearance of glucose in the bloodstream following eating, which is a regular physiological event that depends on the rate of glucose entry into the circulation, the amount of glucose absorbed, the rate of disappearance from the circulation due to tissue uptake, and hepatic regulation of glucose release [94]. Consequently, GI provides information on the GR that might be expected when a person eats food containing CHOs. Glycemic load (GL) considers GI and the total CHO content of a food (GL = GI × available CHO in a given amount of food) [95].

In applying these concepts, foods have been classified by GI into low (GI ≤ 55), medium (GI 56–69), and high (GI ≥ 70) categories, and classified by GL as being low (GL ≤ 10), medium (GL 11–19), and high (GL ≥ 20) [96]. Foods with a low GI value (55 or less) are more slowly digested, absorbed, and metabolized, and cause a lower and slower rise in blood glucose and, therefore, insulin levels. On average, refined grain products and potatoes have a higher GI, legumes and whole grains have a moderate GI, and non-starchy fruits and vegetables have a low GI. Methods of cooking, the physical state of food, dietary protein, fat, and fiber content can affect the GI, which, in turn, can alter rates of glucose absorption [97]. Replacing high GI foods with low GI foods has been shown to improve glycemic control, insulin sensitivity, and HbA_1C_ in patients with T2DM [98,99].

#### 4.1.2. Dietary Fibers

Dietary fibers are complex CHOs, soluble or insoluble, found in plants, that are not hydrolyzed by human enzymes and, therefore, are not digested or absorbed [100]. Insoluble fibers (IF) include cellulose, some hemicellulose, and lignin, while soluble fibers (SF) include mucilage extracted from psyllium husk, gums, pectin, β-glucan, some hemicellulose, and fructans [101]. SF absorb water and form viscous gels capable of increasing food transit time, delaying gastric emptying, decreasing nutrient absorption, slowing digestion, and promoting satiety. SF are found in vegetables (broccoli, onion, carrots, artichokes), fruits (bananas, apples, pears, and berries), legumes, oats, and barley. IF’s food sources are nuts and seeds, wheat, whole grain, bran, some fruits, and vegetables [102]. In addition, some type of SF (β-glucan, arabinoxylans, and pectins) can be easily fermented by the gut microbiota, thus providing a source of short-chain fatty acids, which have been demonstrated to improve glucose control by several mechanisms (reducing the gastric emptying, modifying the release of digestion-related hormones, inhibiting the amylase activity) [103].

Patients with T2DM should consume the amount of dietary fibers generally recommended by the Dietary Guidelines for Americans 2020–2025: a minimum of 14 g of fiber/1000 kcal [68]. Reynolds et al., in their meta-analysis, including 2 prospective cohort studies (8300 adults with T1DM or T2DM residing in 22 countries followed for a mean duration of 8.8 years) and 42 controlled trials (1789 participants with prediabetes, T1DM, or T2DM), demonstrated that an increase in dietary fibers by 1 to 45 g per day improved glycemic control, blood lipids, body weight, and inflammation, and was associated with a reduction in premature mortality in patients with T2DM [104]. Moreover, regular intake of adequate dietary fibers was associated with lower all-cause mortality in patients with T2DM, and prospective cohort studies have found dietary fibers consumption is inversely related to the risk of T2DM [104,105,106,107].

#### 4.1.3. Nutritive Sweeteners

Nutritive sweeteners (NS) generally found in foods and beverages are glucose, fructose, and sucrose [108].

Sucrose, the common commercial sugar, is naturally contained in fruits and vegetables, and is often added to many processed foods [109]. It is hydrolyzed in the small intestine to release an equimolar mixture of glucose and fructose and provides 4 kcal/g [110].

Fructose, a monosaccharide contained in sucrose, is found in fruits, some vegetables, honey, and high fructose corn syrup (HFCS) (up to 55% fructose) [111]. Fructose, mainly as HFCS, is commonly used in beverages and processed foods due to its intrinsically greater sweetness and capacity to improve the appearance and texture of baked foods, and it represents a less expensive alternative to sucrose [111]. Fructose, when consumed in large doses, is a lipogenic sugar, as it increases hepatic de novo lipogenesis through several metabolic pathways. This results in selective deposition of ectopic and visceral fat, and thus adverse effects on lipid metabolism, blood pressure, and insulin sensitivity [112]. As a result, patients with T2DM should limit or avoid consuming sugar-sweetened beverages and processed foods (from any caloric sweetener, including HFCS and sucrose) to reduce the risk for weight gain and worsening of cardiometabolic risk profile [88]. A meta-analysis of 16 controlled feeding trials (236 subjects) showed that fructose at doses > 60 g/day or >10% of energy in isocaloric exchange for other CHOs increased serum triglyceride levels in patients with T2DM [113]. The meta-analysis by Livesey and Taylor also demonstrated a consistent triglyceride-raising effect of fructose at high doses (>100 g/day) across different subject types [114]. In contrast, data from a meta-analysis of 18 short-term controlled feeding trials (209 subjects) indicated that isocaloric fructose replacement by other CHOs resulted in clinically significant improvements in glycemic control, equivalent to a ~0.53% reduction in HbA_1c_, without significantly affecting insulin in patients with T2DM. This benefit was seen across a full dose range (20–160 g/day) of fructose [115]. Thus, recommendations about the ideal quantity of dietary fructose remain disputed due to potential metabolic consequences leading to further insulin resistance and obesity [115].

The current dietary debate is about the amounts and thresholds of NS consumption. Recognizing that many foods containing NS, such as vegetables, fruits, and milk, are beneficial for health and help prevent T2DM, the debate focuses on “added sugar” or “free sugars” (glucose, fructose, and sucrose, commonly as refined sugar, corn syrup, or honey) added to foods and meals at home or in food manufacturing. Commercially available fruit juices or jams may contain only fruit sugars or may contain added sugars. Therefore, it is recommended to prefer products that contain only sugars naturally occurring in fruit. In 2015, the World Health Organization limited its NS intake recommendations [116]. Evidence supports the current recommendation to reduce intake of free sugars to less than 10% of total energy intake since excessive NS promotes adiposity and weight gain [117,118].

Table 1 summarises the recommendations for CHO distribution for individuals with T2DM.

#### 4.1.4. Non-Nutritive Sweeteners

Non-nutritive sweeteners (NNS) present high sweetening properties but lower calorie content than NS (sucrose or HFSC) [119]. They are added in several foods (i.e., diet yogurts, desserts, diet beverages, chewing gum) but can also be found also as table-top sweeteners [119]. The U.S. Food and Drug Administration (FDA) has reviewed several types of NNS for safety, and approved them for consumption by the public, including patients with T2DM [120]:

1. Saccharine is 200 to 700 times sweeter than commercial sugar. The acceptable daily intake (ADI) for saccharine is maximum 5 mg/kg/day [121].
2. Acesulfame K is 200 times sweeter than sugar and is often combined with other sweeteners to decrease its bitter aftertaste. Its ADI is 15 mg/kg/day.
3. Sucralose is 600 times sweeter than commercial sugar. Its ADI is set at 5 mg/kg/day.
4. Aspartame is 200 times sweeter than table sugar. It is the only NNS providing energy (4 kcal/g), but a small amount of it is needed to achieve desired sweetness levels due to its high sweetening power. The ADI recommended by the FDA is 50 mg/kg/d. It is a methyl ester of aspartic acid and phenylalanine dipeptide, thus providing aspartic acid, phenylalanine, and methanol when broken down in the intestine. For this reason, the FDA requires any foods containing aspartame to have an informational label statement: “Phenylketonurics: contains phenylalanine”. Patients with phenylketonuria should avoid products containing aspartame.
5. Neotame is closely related to aspartame in the chemical constitution. It is approximately 7.000 to 13.000 times sweeter than table sugar. The recommended ADI is 0.3 mg/kg/d.
6. Stevia is a non-caloric natural sweetener derived from the leaves of Stevia rebaudiana, a plant native to South America [122,123]. Its sweetness potency is 200 to 400 times sweeter than sucrose [122,123].

In conclusion, NNS can be used as substitutes for NS, to reduce the daily intake of CHOs and calories.

#### 4.1.5. Polyols

Polyols are sugar alcohols obtained via the catalytic hydrogenation of monosaccharides. They are naturally contained in some vegetables and fruits, but they are commonly added into candies, chewing gum, and beverages and provide about 2.4 kcal/g. Polyols constitute an interesting alternative to sucrose, since they do not promote tooth decay, and are slowly and incompletely absorbed in the small intestine [124]. Therefore, they can be used to reduce blood glucose rise and for the management of body weight [124,125,126,127,128,129].

To date, the FDA has approved the use of eight polyols for consumption: erythritol, hydrogenated starch hydrolysates, sorbitol, lactitol, isomalt, maltitol, mannitol and xylitol [130]. Nevertheless, polyols are less sweet than sucrose, and a high amount is required to match the degree of sweetness of sucrose [111]. It is worth mentioning that excessive polyol intake (≥20 g) can induce gastrointestinal symptoms and exert laxative effects [130].

### 4.2. Fat

Dietary fats are represented by fatty acids and cholesterol. Fatty acids (FAs) are organic acids with at least one carboxylic group attached to a carbonaceous chain whose links may form single or double bonds [131]. Generally, naturally occurring FAs have an even number (4–28) of carbon atoms. Specifically, FAs are mainly derived from triglycerides and phospholipids, which are the principal components of food fats. FAs are categorized into saturated fatty acids (SFAs), with no double bonds, monounsaturated fatty acids (MUFAs) with one double bond, polyunsaturated fatty acids (PUFAs) with more than one double bond, and trans fatty acids (TFAs) with single or one or more isolated double bonds in a trans configuration. PUFA can be further classified in omega-6 PUFAs and omega-3 PUFAs. This variant depends on the position of the first double bond from the methyl-end of the molecule; moreover, there are also omega-7 and omega-9 PUFAs, which are not essential [131].

Data are uncertain about an ideal total fat intake for patients with T2DM [132]. Indeed, there is no established standard percentage of calories from fat for patients with T2DM, while more attention has been addressed to fat quality [133].

Due to the high risk of cardiovascular disease in patients with T2DM, it is recommended to promote the substitution of SFAs and TFAs with healthier oils and foods higher in unsaturated FAs, such as MUFAs, and PUFAs [133].

#### 4.2.1. Saturated Fatty Acids

SFAs are FAs that have no double bonds. Fats containing more SFAs are typically solid or nearly solid at ambient temperature. SFAs are found in all animal fats such as butter, and some plant oils, such as coconut, palm kernel, and palm oil [134]. In addition, processed and fast foods have an elevated quantity of SFAs. Tropical oils, such as coconut and palm, are usually advertised as beneficial SFAs because they originated from plants, but this is not correct [134]. The American Heart Association advises choosing non-tropical vegetable oils when cooking, and suggests replacing saturated with unsaturated fats, especially PUFAs, which may prevent CVD [134,135].

Nutritional guidelines recommend that patients with T2DM should be advised to follow the guidelines for the general population for the intake of SFAs [33,68]. The consumption of SFAs should not exceed 10% of total calories per day and be substituted with unsaturated fats, particularly PUFAs [134]. Conversely, it has been shown that a diet high in SFAs may increase CVD risk. Substituting SFAs with PUFAs, significantly lowers total cholesterol and LDL-cholesterol (LDL-C), and substituting SFAs with MUFAs from plant sources, for example, olive oil and nuts, decreases CVD risk [134,135]. Likewise, substituting SFAs with CHOs lowers total cholesterol and LDL-C, even if it significantly raises triglycerides and reduces HDL-cholesterol (HDL-C) [88]. In addition, switching SFAs with CHOs does not reduce the risk of CVD [134,135]. 

A recent systematic review and meta-analysis including 5 RCTs (for a total of 22,591 participants, with or without TD2M) showed that the substitution of SFAs with PUFAs or CHOs is correlated with a decrease in cardiovascular events [136]. Another recent meta-analysis comprising 15 RCTs (56,675 participants) suggested that decreasing SFA consumption for at least 2 years triggers a possible significant reduction in cardiovascular events, asserting that the transition from SFAs to PUFAs or CHOs appears to be a helpful approach, while the consequences of substitution with MUFAs are uncertain [137]. In summary, it is advisable to reduce the consumption of SFAs to no more than 10% of total daily caloric intake and substitute them with unsaturated fats.

#### 4.2.2. Trans Fatty Acids

TFAs are fats generated when oils are “partially hydrogenated” [132]. According to the FDA, TFAs are defined as the amount of all unsaturated FAs that include one or more isolated (non-conjugated) double bond in a trans configuration. The European Food Safety Authority (EFSA) provides a distinct classification of TFAs, to distinguish them as trans-MUFAs (that are the most common TFAs in the human diet) or trans-PUFAs (composed by as a minimum one trans double bond, and thus could additionally have double bonds in the cis configuration) [138]. The EFSA reported that TFAs might derive from various sources. However, a first classification distinguishes between natural (also called “ruminant”) and artificial (or “industrially produced”) sources [138]. The first include the bacterial conversion of unsaturated fatty acids into TFAs in the rumen of ruminants (such as cattle and sheep) that produce dairy products, while the second include industrial hydrogenation, a procedure that shifts the chemical composition of unsaturated fats by inserting hydrogen atoms. This technique is used by manufacturers to produce semi-liquid and solid fats, which enhances product steadiness and shelf-life, and hence it is less likely to become spoiled or rancid. 

Several studies showed that increased consumption of industrial TFAs is associated with a higher risk of CVD. Indeed, industrial TFAs increase the circulating concentrations of total and LDL-C and reduce HDL-C concentrations, resulting in a stronger atherogenicity compared with SFAs or cis-unsaturated FAs. Moreover, they may promote atherogenesis by activating inflammation and reactive oxygen species production and injuring endothelial cells [139]. A meta-analysis of 7 RCTs studies (208 subjects), showed that TFA intake did not result in significant changes in glucose and insulin concentrations, but the increase in TFA intake led to a significant increase in total and LDL-C (3.1%) [140].

Therefore, TFA intake should be avoided; the recommended intake is less than 1% of total energy.

#### 4.2.3. Monounsaturated Fatty Acids

MUFAs are FAs with one double bond and are usually liquid at room temperature. MUFAs are also called omega-9, as their only unsaturation is at position 9 concerning the methyl terminal; for reference, there are also omega-7 MUFAs [141]. The most common dietary MUFA is oleic acid, found primarily in olive oil, followed by high oleic-sunflower and safflower oil, and canola oil. MUFAs are also present in avocado, some fatty fish, nuts, and nut-based butters [141].

MUFAs have been shown to improve insulin sensitivity by inhibiting endoplasmic reticulum (ER) stress and anti-inflammatory activities. Moreover, they enhance β cell survival and inhibit the reduction of the insulin signaling pathway [142].

A meta-analysis of 24 RCTs, evaluating diets rich in MUFAs vs. CHOs or PUFAs, showed that high-MUFA-containing diets might improve metabolic factors in patients with T2DM, i.e., fasting insulin and glucose level, homeostasis model of assessment for insulin resistance (HOMA-IR) and β-cell function, HDL-C levels, and triglyceride levels [143].

There are currently no daily limits on MUFAs intake for the general population or patients with T2DM, except recommendations to maintain total fat intake between 25% to 35% of total daily calories and to prefer foods that contain MUFAs or PUFAs instead of SFAs or TFAs [133].

#### 4.2.4. Polyunsaturated Fatty Acids

PUFAs are fatty acids with two or more double bonds and are usually liquid at room temperature. PUFAs provide essential fats, such as omega-3 and omega-6 FAs, found in significant amounts in sunflower, corn, soybean, and cottonseed oils, walnuts, pine nuts, sesame, pumpkin, and flax seeds. Only a small amount of PUFA is found in animal fats 

Recently, a meta-analysis of 102 RCTs (4660 participants) showed different results regarding macronutrients’ effects on glucose–insulin homeostasis and concluded that the most compelling positive effects were observed for PUFAs compared to CHOs, MUFAs, or SFAs, as PUFA replacement resulted in improved blood glucose levels, insulin resistance, and insulin secretion capacity [144]. Another meta-analysis comprising 15 RCTs (667 participants) reported that the consumption of walnuts (a good source of PUFAs) prevented the onset of T2DM and improved blood glucose levels [145]. Furthermore, the intake of pistachios for three months also significantly reduced triglycerides (mean difference [MD] −0.28 mmol/L), and other nuts (almonds and hazelnuts) reduced fasting blood sugar and glycated hemoglobin (MD −0.26 mmol/L and −0.11% respectively) [145]. However, a systematic review and meta-analysis including 83 RCTs assessed the effects of increased PUFAs on glucose metabolism in patients with T2DM over 24 weeks, which demonstrated that long-chain omega-3 PUFAs have minimal or no effect on glucose metabolism [14]. Moreover, harmful outcomes might occur when the amount of supplemental long-chain omega-3 PUFAs exceeds 4.4 g/day [14].

Regarding omega-6 PUFAs, current data show that linoleic acid (LA) consumption is frequently lower than the recommended amounts. Therefore, there are currently no daily limits on omega-6 PUFA intake for the general population or patients with T2DM.

#### 4.2.5. Omega-3 PUFAs

Omega-3 polyunsaturated fatty acids (PUFAs) include α-linolenic acid (ALA; 18:3 ω-3), stearidonic acid (SDA; 18:4 ω-3), eicosapentaenoic acid (EPA; 20:5 ω-3), docosapentaenoic acid (DPA; 22:5 ω-3), and docosahexaenoic acid (DHA; 22:6 ω-3). Since humans do not have the enzymes necessary to produce omega-3 PUFAs, they are deemed essential FAs, so they must be taken from the diet [146,147]. The form of omega-3 PUFAs in plants is alpha-linolenic acid (ALA, 18:3), and the primary sources include soybean oil, canola oil, walnuts, flaxseed, and chia seeds. The animal sources of omega-3 are eicosapentaenoic acid (EPA, 20:5) and docosahexaenoic acid (DHA, 22:6), which are very-long-chain n-3 FAs contained in seafood, such as fish and shellfish. Fish with a high level of omega-3 include salmon, albacore tuna, mackerel, sardines, herring, and lake trout [134]. At the metabolic level, ALA can be transformed into both DHA and EPA, but this reaction’s rate is very low, indicating the need for direct food intake of DHA [147,148]. Omega-3 PUFAs reduce triglycerides, which are additional risk factors for CVD, and are usually dysregulated in patients with T2DM. The mechanism of action of omega-3 PUFAs on lowering triglycerides is not completely identified yet but is supposed to inhibit the expression of lipogenic genes, improve beta-oxidation of FAs, and enhance the expression of lipoprotein-lipase (LPL) [149,150,151]. Additional mechanisms of action may explain the potentially beneficial properties of omega-3 PUFAs on T2DM. Specifically, omega-3 PUFAs can regulate numerous inflammatory pathways, including the suppression of pro-inflammatory cytokines (such as TNF-alpha, IL-1, IL-6), the suppression of cyclo-oxygenase function (and its consequent eicosanoid production from arachidonic acid, such as leukotrienes and prostaglandins), the suppression of pro-inflammatory transcription nuclear factor kappa B (NF-ĸB), and the activation of peroxisome proliferator-activated receptors (PPARs) [152,153,154,155].

However, data on the effect of omega-3 PUFAs from food in patients with T2DM are insufficient and inconclusive. A recent meta-analysis suggested an association between reduced incidence of T2DM and a higher intake of vegetable fats, particularly of ALA of vegetable origin, up to a maximum of 560 mg per day, and lower amounts for total PUFAs [16]. In contrast, long-chain omega-3 PUFAs of animal origin were related to an increased incidence of T2DM, although geographic differences were found [16].

Previous data did not consistently support recommending omega-3 PUFA (EPA and DHA) supplements in patients with T2DM to prevent or manage CVD [88]. In fact, in the ASCEND trial (carried out on 15,480 patients with T2DM and no CVD), the use of omega-3 PUFAs (1 g/day) did not bring cardiovascular advantages [156]. Similar findings were reported in the VITAL trial (in which 13% of the 25,871 participants were patients with T2DM), as the supplementation with 1 g of omega-3 PUFAs did not decrease the frequency of major cardiovascular outcomes [157]. Nevertheless, in the REDUCE-IT trial (in which 57% of the 8179 participants were patients with T2DM), 2 g of prescribed icosapentethyl twice a day significantly decreased cardiovascular outcomes by 25% [158].

Nonetheless, at present the FDA has authorized the use of two omega-3 PUFA-containing products: omega-3-acid ethyl esters (icosapent ethyl) and omega-3-carboxylic acids [146]. The supplements mentioned above are approved for adults (≥18 years of age) with hypertriglyceridemia (≥500 mg/dl) in combination with diet to decrease triglyceride levels and reduce the risk of cardiovascular events [159,160,161]. Furthermore, the FDA advises that the total daily consumption of omega-3 PUFAs should not exceed 3g of combined EPA and DHA per day, with no more than 2 g/day taken from supplementation [146].

#### 4.2.6. Dietary Cholesterol

Cholesterol is a natural sterol present in all animal tissues, and it is a waxy fat-like substance. Cholesterol derives from an external source through food and an internal source through its endogenous synthesis by the liver and extrahepatic tissues. Furthermore, it is the precursor of steroid hormones and an essential component of the cell membranes. Cholesterol circulates into the bloodstream through molecular aggregates called lipoproteins. HDL-C carries cholesterol from the tissues to the liver, while LDL-C goes in the opposite direction, transporting cholesterol from the liver to the tissues. Food sources of cholesterol are egg yolks, chicken, pork, beef burgers, and cheese [162]. It is absent in plant products such as fruit, vegetables, and cereals [162].

Nutritional guidelines recommend consuming no more than 300 mg/day of cholesterol from food sources, a limit that drops to 200 mg/day if the patients have a high risk of developing CVD [33]. Usually, the intake of dietary cholesterol is associated with a greater intake of SFAs, which, in turn, can increase LDL-C levels with a consequent risk of CVD [136]. However, a meta-analysis including six studies of TD2M patients, suggested no correlation between dietary cholesterol (contained in eggs) and cardiovascular disease in T2DM patients [163]. Therefore, there is no solid evidence for the limitation of egg consumption that was imposed in the past. Nevertheless, low-fat dairy products should be consumed in order to limit the total cholesterol intake [163].

Table 2 summarises the recommendations for dietary fats distribution for individuals with T2DM.

### 4.3. Protein

Proteins are molecules made up of amino acids, linked to one another by peptide bonds in long chains. There are 20 different amino acids that can be combined to form every type of protein in the body. These amino acids fall into two major categories: essential amino acids, obtained from food, and non-essential amino acids, synthesized by cells [164]. Proteins are found in plant and animal foods, such as legumes, dairy products (milk, cheese, and yogurt), eggs, meats, poultry, nuts, seafood (fish and shellfish), soy products, whole grains, and vegetables [164].

Many studies focus on the amount of dietary protein and their relationship with glycemic control in patients with T2DM. A randomized crossover study on eight patients with T2DM showed that a high-protein-low-CHO diet improves glycemic control with statistically significant results [165]. A recent cross-sectional study of 212 participants (most participants were between the ages of 45 and 75, overweight or obese, with prediabetes or T2DM) suggested that the mean value of HOMA-IR is lower in those with a protein intake higher than recommendations as compared to those whose protein intake was lower [166]. A meta-analysis of 9 short-term studies (418 subjects with T2DM) showed that high-protein diets resulted in a better glycemic control (decreased HbA1c by 0.52%) and weight loss but did not affect fasting blood glucose or lipid levels [167].

However, there is limited evidence that adjusting the daily level of protein intake (typically 1–1.5 g/kg body weight/day or 15–20% of total calories) may improve glycemic goals in patients with T2DM. A meta-analysis by Dong and colleagues found successful management of T2DM with a moderately higher level of proteins, at 20–30% of total calories, which may increase satiety in these patients [167].

Additionally, replacing animal with plant proteins could confer some glycemic control benefits in patients with T2DM. A meta-analysis of 13 RCTs (280 middle-aged adults) showed that replacing animal with plant proteins led to modest improvements in glycemic control in patients with T2DM, as the HbA1c, fasting glucose, and fasting insulin were decreased in diets that replaced the animal with plant proteins at a median level of ~35% of total protein per day [168]. A potential explanation could be the reduction of heme iron intake, found only in animal protein sources, which is significantly associated with an increased risk of T2DM, as opposed to non-heme iron intake, found both in plant and animal foods, which is inversely associated with or not associated with T2DM incidence [168].

Moreover, the daily intake of proteins for patients with T2DM should be individualized. Those with diabetic kidney disease need to restrict protein intake < 0.8 g/kg of body weight [169]. The National Kidney Foundation recommends maintaining the daily protein intake at no more than 0.8 g/kg of body weight for patients with T2DM who have kidney complications, while a meta-analysis of the Cochrane Database advises a protein intake of 20–30% of total calories for people without kidney complications [170].

Table 3 summarises the recommendations for dietary proteins distribution for individuals with T2DM.

## 5. Micronutrients

Despite T2DM being associated with increased oxidative stress, current studies report no benefit of routine supplementation with antioxidants, such as carotene, or vitamins E and C, due to lack of evidence showing efficacy and long-term safety [68]. In addition, scientific evidence does not support the regular use of herbal supplements and micronutrients, such as curcumin, cinnamon, aloe vera, vitamin D, zinc, magnesium, or chromium, to improve glycemia or treat diabetes complications in patients with T2DM without a documented deficiency [68]. A multivitamin supplement may be necessary for special populations, including pregnant or lactating women, older adults, patients with celiac disease, vegetarians, and people following very-low-calorie or low-CHO diets [88].

Blood testing of vitamin B12 levels should be considered in metformin-treated patients, particularly those with anemia or peripheral neuropathy [171]. The exact cause of B12 deficiency in patients taking metformin is unclear. The standard of care is B12 injections, but some studies suggest that high-dose oral supplementation may be as effective [172,173].

## 6. Fluid Intake

Recommendations about total daily fluid intake are less common and accurate than food recommendations. Increasing water intake during a meal could contribute to a sense of fullness, thus decreasing energy intake [174,175,176]. Moreover, replacing diet beverages (NNS beverages) or sugar-sweetened beverages with water is an effective goal for overweight/obese patients with T2DM in order to improve weight loss and glycemic control, since water contains zero calories [176,177]. It is imperative to conduct more extensive and more rigorous trials to answer whether drinking water can improve glycemic parameters in patients with T2DM, as there is lack of proper evidence in this regard. It is, however, reasonable to recommend a water intake similar to the general population.

Regarding alcohol consumption, nutritional guidelines recommend no more than one drink/day for women and two drinks/day for men, as for the general population (one drink refers to 12 oz = 341 mL beer, 5 oz = 142 mL glass of wine, or 1.5 oz= 43 mL of distilled spirits) [178]. Even though moderate alcohol consumption may have cardiovascular benefits, alcohol intake may increase the risk for delayed hypoglycemia in patients with T2DM, due to its effect of preventing the liver from releasing glucose. This is particularly relevant for patients using insulin or insulin secretagogues, as they can experience delayed nocturnal or fasting hypoglycemia after evening alcohol consumption. The risk of hypoglycemia can be minimized by consuming alcohol with food. 

In addition, weekly alcohol consumption has been associated with an increased risk for death, disability, and social problems, mainly triggered by cancers, liver disease, unintentional injuries, and violence. Finally, it is worth mentioning that alcohol consumption (2 drinks/week) can increase the risk for some cancers, especially breast and colon [178].

Therefore, it is important that patients with T2DM are advised about a low-to-moderate and sensible alcohol consumption for people choosing to consume alcohol, considering also individual tolerance [88]. People should not be encouraged to start drinking alcohol. 

## 7. Nutraceuticals and Supplements

### 7.1. Phytosterols

Phytosterols are a group of chemical compounds of plant origin, including stanols, sterols, and esters. Structurally, phytosterols are like cholesterol, but they are poorly absorbed in the gut [179]. They are not synthesized in humans and therefore are introduced exclusively through the diet. Phytosterols are present in dried fruit such as almonds but also in vegetable oils, mainly corn oil, sunflower oil, soybean oil, and olive oil, and in cereals (wheat germ and wheat bran), and fruits and vegetables (cauliflower, passion fruit, and orange) [180].

Phytosterols can help manage hypercholesterolemia, particularly LDL-cholesterol concentrations, by reducing intestinal cholesterol absorption in the intestine through several mechanisms [181,182,183]: (1) competition with cholesterol through solubilization in mixed micelles in the intestinal lumen; (2) reduction of cholesterol esterification in the enterocyte; (3) reduction of cholesterol through trans-intestinal excretion; (4) modification of gene expression encoding sterol-carrying proteins, such as the Niemann-Pick C1-like 1 (NPC1-L1) protein, reducing the transport of cholesterol to the enterocyte.

The proportion of phytosterols in certain foods cannot exert a therapeutic effect. Thus, many food items (i.e., yogurt, cereals, and margarine) are fortified with phytosterols. Indeed, the cholesterol-lowering effect is dose-dependent for phytosterol intake below 3 g/day. Notably, above this dose no further reduction in LDL cholesterol is observed [179]. However, few studies have analyzed the long-term effects in patients with T2DM treated with phytosterols. A meta-analysis of 5 RCT (n: 148 individuals with T2DM) reported a significant LDL-cholesterol reduction (−12 mg/dL), with no effect on HDL fraction [184].

Regarding the cholesterol-lowering effect, an RCT carried out on 200 patients with impaired glucose regulation showed that phytosterols (3 g/day) can significantly reduce triglyceride concentrations and inflammation (measured by c-reactive protein) [185].

Nevertheless, an excessive intake of phytosterols may reduce the absorption of fat-soluble vitamins. In addition, individuals with heterozygous sitosterolemia (a rare autosomal recessive disease characterized by phytosterol accumulation due to ABCG5 or ABCG8 gene mutations) should not take phytosterols. Meanwhile, others can tolerate the intake of phytosterols with no occurrence of health problems such as hypercholesterolemia, early atherosclerosis development, or increased cardiovascular morbidity and mortality. However, the threshold of safe intake has not yet been defined [179]. According to the available evidence, no side effects for regular consumption of 2 g/day of phytosterols have been recorded.

Therefore, phytosterols supplementation (above 3 g/day) could be advised under medical supervision to patients with T2DM to reduce LDL-cholesterol concentrations. 

### 7.2. Polyphenols

Polyphenols are one of the largest family of phytochemical compounds found in nature [186]. They can be found in a variety of plant foods, such as berries, fruits and citrus fruits, green leafy vegetables, spices and herbs, green tea and coffee, olive oil, and nuts. In addition, they can be easily supplemented by adding their extracts into food items or pills [186]. 

Polyphenols have an ample diversity of chemical structures, with at least one or more phenolic groups; they typically present antioxidant, anti-inflammatory, neuroprotective, and anti-obesity/weight-reducing effects [187,188]. As for their role in the management of T2DM, polyphenols have been shown to hamper CHO digestion in the intestine, decreasing glucose absorption and influencing glucose metabolism [189]. They can also enhance insulin sensitivity and β cell function [186]. Furthermore, it has been reported that they control the islet amyloid polypeptide (IAPP) or amylin secretion [190].

Nevertheless, these effects have been demonstrated only for some classes of polyphenols.

Resveratrol is one of the most studied phenolic compounds. It belongs to the stilbenes subclass [191]. Several meta-analyses of RCTs in patients with T2DM or pre-diabetes have reported a dose–response effect of resveratrol (≥500mg/day) in the improvement of outcomes related to glucose metabolism (fasting glucose and insulin, HbA1c) [192,193,194], cholesterol and triglyceride concentrations [195,196], and blood pressure [197].

Quercetin, one of the most common flavonols, has been shown to reduce hyperglycemia and its associated micro- and macrovascular complications [191,198]. As recently summarized by Ansari and colleagues, in vitro and animal models demonstrated that quercetin could reduce oxidative damage and inflammation in the pancreatic beta cells and in other tissues [198]. In addition, it can influence GLUT2 expression and sodium-dependent glucose uptake in the gut, thus reducing glucose absorption. However, few clinical studies have been carried out with inconclusive results [198]. 

Oleuropein complex and phenolic alcohols (tyrosol and hydroxytyrosol) are found in olive derivatives (fruits, leaves, branches, and oil). Olive extract supplementation has been shown to influence glucose metabolism in healthy subjects as well as in individuals with type 2 diabetes [199]. These effects are mainly related to the improvement of insulin secretion, likely due to the increase of glucagon-like peptide-1 (GLP-1). Notably, olive oil polyphenols (5 mg/day) bear a health claim by the EFSA for the protection of LDL particles from oxidative damage [200].

Among other polyphenols, anthocyanins and flavan-3-ols have been shown to significantly improve insulin sensitivity and lipid metabolism [201,202] whereas catechins can influence weight loss and maintenance, likely through increased oxidation in adipocytes, inhibition of lipogenesis, and increased energy expenditure [203]. In addition, catechins, in particular epigallocatechin gallate, have been appointed a sharp antioxidant activity that reduces oxidative stress, endothelial dysfunction, and consequent inflammation [204].

It is worth mentioning that cocoa polyphenols (200 mg of flavan-3-ols, 2,5 g cocoa powder, or 10 g of dark chocolate) bear a health claim by the EFSA for the maintenance of normal endothelium-dependent vasodilation [200].

Despite these promising results, the current evidence does not allow establishing the dose of polyphenols or specific phenolic compounds to be recommended for the management of T2DM.

Indeed, the studies available so far present several methodological limitations. First, duration and dose of polyphenol supplementation in very heterogeneous among the studies. Furthermore, no information is provided on the bioavailability of some phenolic compounds when they are ingested as food items or extracts.

Nevertheless, the daily consumption of naturally polyphenol-rich food items might be advised as part of a healthy diet according to the current nutritional dietary recommendation.

### 7.3. Inositol

Inositol is a carbocyclic sugar that can be synthesized by both prokaryotic and eukaryotic cells. In mammals, it is obtained from dietary sources (as free inositol, phosphatidyl-inositol or inositol-6-phosphate) or the endogenous synthesis from glucose [205].

The main dietary sources are plant foods, such as soybean and legumes, cereals (in particular buckwheat), and pumpkin [206]. Inositol has nine stereoisomers, but the compounds with biological activity are myo-inositol and D-chiro-inositol [207].

Dietary inositol is mainly in the form of myo-inositol, and after intake it is converted to D-chiro-inositol by an NAD–NADH-dependent epimerase, under the stimulus of insulin. Under physiological conditions, the myo-inositol/D-chiro-inositol ratio is 40:1 in plasma [207].

All cell membranes have inositol phospholipids, which are part of intracellular messengers, and they can modulate the members of insulin signaling pathways [208]. In particular, inositols are second messengers that favor the translocation of GLUT4 (glucotransporter 4) and glucose uptake in the cells [208]. In addition, D-chiro-inositol can increase glycogen synthesis and storage in the liver or skeletal muscle. Finally, D-chiro-inositol can influence the expression of key protein participating in insulin and other hormone signal transduction (i.e., IRS2, PI3K, and AKT) [206]. Therefore, inositols have been used as insulin sensitizers in many clinical settings characterized by the occurrence of insulin resistance, i.e., metabolic syndrome, PCOS, GDM, and other individuals with any abnormal glucose homeostasis [205,207].

A recent meta-analysis reported that the supplementation with myo-inositol (1–4 g/day) in 1319 pregnant women significantly reduced the incidence of GDM [209]. However, the studies presented a huge variability in doses, duration, and timing of administration of myo-inositol [209].

As for the evidence in patients with T2DM, a meta-analysis considering 20 RCTs (n= 1239 individuals with T2DM or prediabetes) showed that the supplementation with inositol (1.2–4 g/day) improved fasting blood glucose and insulin sensitivity (HOMA-IR), independently of body weight loss [210].

Notably, no serious side effects have been reported for the use of inositol in these studies. Mild gastrointestinal side effects (i.e., nausea, flatus, and diarrhea) have been reported in studies with higher doses of myo-inositol (12 g/day) [211].

In conclusion, the studies available so far suggest that inositol can improve glucose metabolism, likely increasing insulin sensitivity. However, further studies are needed to establish the most effective combination of inositol isomers (myo-inositol and D-chiro-inositol) to formulate a clear recommendation for its use in the management of T2DM.

### 7.4. Microalgae

Microalgae are unicellular microorganisms located in saline and freshwater. They are rich in proteins (phycobiliproteins), CHOs (polysaccharides), lipids (ω-3 PUFAs, sterols), nucleic acids, and many bioactive components such as dietary fiber, antioxidants (polyphenols, tocopherols), and pigments (carotenoids, phycocyanine, chlorophylls, xanthophylls) [212,213,214]. Even though there is a vast variety of microalgae, the ones that have been approved by the EFSA to be used for human nutrition are Arthrospira (also known as Spirulina), Chlorella, and Tetraselmis [214]. Edible microalgae have a particular structure that might affect insulin resistance onset and pancreatic cell function [214]. ω-3 PUFAs and carotenoids, by their antioxidant and anti-inflammatory properties, help to compensate for systemic inflammation and pancreatic impairment. Microalgae’s prebiotic polysaccharides contribute to conserving the intestine’s integrity, and its phenolics may have a part by directly impacting gut microbiota, or concomitantly by the intervention of the endogenous antioxidant defense system [214]. In this respect, a meta-analysis of 7 clinical and 27 preclinical studies, about the effects of Arthrospira (Spirulina) supplementation on glucose metabolism and glycemic control showed that in clinical studies it decreased fasting blood glucose, triglycerides, and total cholesterol levels, and increased HDL-C for patients with T2DM, but it did not have an impact on HbA1c, while in animal studies it decreased fasting blood glucose and HbA1c [215]. However, further studies are needed to fully elucidate their potential benefit on glucose control in T2DM.

### 7.5. Berberine

Berberine, an alkaloid component obtained from roots and rhizomes, has a variety of therapeutic applications for inflammation, infections, and diabetes [216,217]. Regarding the latter, it has been demonstrated that berberine increases insulin sensitivity and insulin expression, decreases blood insulin levels, stimulates beta cell regeneration, has antioxidant enzyme activity, and reduces lipid peroxidation [216,217,218]. A recent systematic review (18 RCTs) evidenced that berberine decreased HOMA-IR and fasting plasma glucose, and improved lipid profile [217]. Another meta-analysis of 28 RCTs (2313 patients with T2DM) demonstrated that the use of berberine was related to better blood glucose control, including HbA1c, and that its combination with oral hypoglycemic agents provided a more significant reduction of glycemia than using each of them alone [219]. Nevertheless, almost all studies with berberine supplementation have been performed in Asian populations, making difficult the transferability of the results to other populations. In addition, no information on the bioavailability of berberine among different formulations is available.

Therefore, there is still the need for more evidence about the efficacy and safety of berberine.

## 8. Conclusions

Individualized medical nutritional therapy, as part of lifestyle optimization, is the cornerstone for the management of T2DM and has been shown to improve metabolic outcomes.

To date, scientific evidence does not support a specific macronutrient distribution and meal plans should be individualized. Nevertheless, reducing the overall CHO intake and replacing high GI foods with low GI foods have been shown as valid options for patients with T2DM to improve glycemic control. In addition, the intake of free sugars must not exceed the 10% of total energy intake to avoid hyperglycemia and weight gain. The substitution of saturated and trans fatty acids for monounsaturated and polyunsaturated fats reduces cardiovascular risk and improves glucose metabolism.

**Figure 1:**
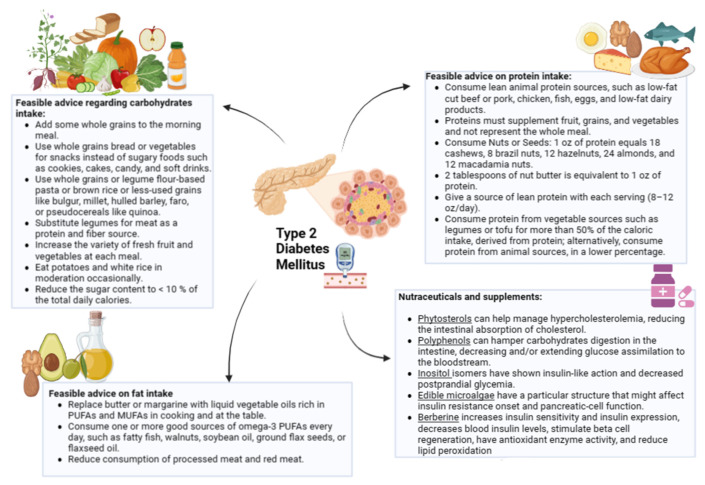
Graphic representation of dietary and integrative treatment for patients with type 2 diabetes mellitus. PUFAs, polyunsaturated fatty acids; MUFAs, monounsaturated fatty acids.

As for antioxidant supplementation (i.e., carotene, vitamins E and C) and nutraceuticals (i.e., phytosterols, polyphenols, microalgae, inositols, and berberine), further studies are needed to evaluate the efficacy and the long-term safety in patients with T2DM. The key practical advice is summarized in Figure 1.

## Figures and Tables

**Table 1:** Recommended CHO distribution for patients with T2DM.

Type of CHO	Main Source	Recommended Daily Dose	References
Total CHO	Vegetables, legumes, fruit, dairy (milk and yogurt), and whole grains	44–46% of total daily calories (depending on individual goals, it can be 26–45% of total calories, or less than 26% of total calories)	[88,93]
Dietary Fibers	Fruit, vegetables, nuts, seeds, wheat, whole grain, bran	14 g of fiber/1000 kcal minimum	[68]
Nutritive Sweeteners	Fruit, some vegetables, honey, HFCS, sugar-sweetened beverages, and many processed foods	<10% of total daily calories	[116,118]

Abbreviations: CHO, carbohydrates; T2DM, type 2 diabetes mellitus; HFCS, high fructose corn syrup.

**Table 2:** Recommendation regarding dietary fat intake for patients with T2DM.

Type of Fat	Main Source	Recommended Daily Dose	References
Total Fats		25–35% of the total daily calories	[82]
MUFAs	Canola, peanut, and olive oils; avocados; nuts such as almonds, hazelnuts, and pecans; and seeds such as pumpkin and sesame seeds	There are currently no daily limits on MUFA intake for the general population or patients with T2DM	[135]
Omega-6	Sunflower, corn, soybean, cottonseed, and flaxseed oils, pine nuts, sesame, pumpkin, and in foods such as walnuts, flax seeds, and fish	There are currently no daily limits on omega-6 PUFAs intake for the general population or patients with T2DM	-
Omega-3	Seafood, such as fish and shellfish, including salmon, albacore tuna, mackerel, sardines, herring, and lake trout Soybean oil, canola oil, walnuts, flaxseed, and chia seeds;	Daily consumption does not surpass 3g/day of EPA and DHA overall, with no more than 2g/day taken from supplementation	[147]
SFAs	Whole milk, butter, cheese, ice cream, red meat, chocolate, coconuts, coconut milk, coconut oil, and palm oil	Less than 10% of the total calories from the diet	[68,82,134]
TFAs	Some margarines, partially hydrogenated vegetable oil; deep-fried foods, many fast foods, some commercial baked goods, dairy products, beef, and lamb	Less than 1% of total calories from the diet, 2 g/day maximum	[82,132,141]
Cholesterol	Eggs, chicken, beef, burgers, cheese	300 mg/day maximum, 200 mg/day for people with a high risk of CVD	[33,82]

Abbreviations: MUFA, monounsaturated fatty acid; T2DM, type 2 diabetes mellitus; PUFA, polyunsaturated fatty acid; EPA, eicosapentaenoic acid; DHA, docosahexaenoic acid; SFA, saturated fatty acids; TFA, trans fatty acid; CVD, cardiovascular diseases.

**Table 3:** Recommendations regarding dietary proteins for patients with T2DM.

Type of Nutrient	Main Source	Recommended Daily Dose	References
Protein	Animal: dairy products (milk, cheese, and yogurt), eggs, meat, poultry, seafood (fish and shellfish) Plant-based: legumes, nuts, tofu, seeds	20–30% of total daily calories	[170]

## Data Availability

No new data were created or analyzed in this study. Data sharing is not applicable to this article.
